# Altered expression of vesicular glutamate transporter-2 and cleaved caspase-3 in the locus coeruleus of nerve-injured rats

**DOI:** 10.3389/fnmol.2022.918321

**Published:** 2022-07-27

**Authors:** Lidia Bravo, Patricia Mariscal, Meritxell Llorca-Torralba, Jose María López-Cepero, Juan Nacher, Esther Berrocoso

**Affiliations:** ^1^Neuropsychopharmacology and Psychobiology Research Group, Department of Neuroscience, University of Cádiz, Cádiz, Spain; ^2^Instituto de Investigación e Innovación Biomédica de Cádiz, INiBICA, Hospital Universitario Puerta del Mar, Cádiz, Spain; ^3^Centro de Investigación Biomédica en Red de Salud Mental (CIBERSAM), Instituto de Salud Carlos III, Madrid, Spain; ^4^Neuropsychopharmacology and Psychobiology Research Group, Department of Psychology, University of Cádiz, Cádiz, Spain; ^5^Neuropsychopharmacology and Psychobiology Research Group, Department of Cell Biology and Histology, University of Cádiz, Cádiz, Spain; ^6^Neurobiology Unit, Program in Neurosciences and Institute of Biotechnology and Biomedicine (BIOTECMED), Universitat de València, Burjassot, Spain; ^7^Fundación Investigación Hospital Clínico de Valencia, INCLIVA, Valencia, Spain

**Keywords:** locus coeruleus, vesicular glutamate transporter 1, vesicular glutamate transporter 2, cleaved caspase 3, neuropathic pain

## Abstract

Neuropathic pain is a debilitating chronic condition provoked by a lesion in the nervous system and it induces functional alterations to the noradrenergic locus coeruleus (LC), affecting distinct dimensions of pain, like sensorial hypersensitivity, pain-induced depression, and anxiety. However, the neurobiological changes induced by nerve damage in the LC remain unclear. Here, we analyzed excitatory and inhibitory inputs to the LC, as well as the possible damage that noradrenergic neurons suffer after the induction of neuropathic pain through chronic constriction injury (CCI). Neuropathic pain was induced in male Sprague-Dawley rats, and the expression of the vesicular glutamate transporter 1 or 2 (VGLUT1 or VGLUT2), vesicular GABA transporter (VGAT), and cleaved caspase-3 (CC3) was analyzed by immunofluorescence 7 (CCI7d) or 28 days after the original lesion (CCI28d). While no significant differences in the density of VGLUT1 puncta were evident, CCI7d induced a significant increase in the perisomatic VGLUT2/VGAT ratio relative to Sham-operated and CCI28d animals. By contrast, when the entire region of LC is evaluated, there was a significant reduction in the density of VGLUT2 puncta in CCI28d animals, without changes in VGLUT2/VGAT ratio relative to the CCI7d animals. Additionally, changes in the noradrenergic soma size, and a lower density of mitochondria and lysosomes were evident in CCI28d animals. Interestingly, enhanced expression of the apoptotic marker CC3 was also evident in the CCI28d rats, mainly co-localizing with glial fibrillary acidic protein but not with any neuronal or noradrenergic marker. Overall, short-term pain appears to lead to an increase of markers of excitatory synapses in the perisomatic region of noradrenergic cells in the LC, an effect that is lost after long-term pain, which appears to activate apoptosis.

## Introduction

Neuropathic pain is a debilitating chronic pain condition that arises after the nervous system is damaged, and it is associated with a plethora of changes in the peripheral and central nervous systems (PNS and CNS). Indeed, functional plasticity has been reported after nerve injury in the noradrenergic locus coeruleus (LC) that affects all dimensions of pain, including its long-term affective consequences on depression and anxiety. The LC is located on the floor of the fourth ventricle in the rostral pons, and it provides and receives extensive projections throughout the neuroaxis, representing a critical hub for pain neurotransmission (Llorca-Torralba et al., 2016; Suarez-Pereira et al., 2021). It has been demonstrated that lesions and pharmacological or chemogenetic inactivation of the LC exacerbate pain responses soon after nerve injury, suggesting enhanced pain sensitivity when the normal LC-noradrenergic circuit is disrupted (Bodnar et al., 1978; Martin et al., 1999; Camarena-Delgado et al., 2021; Llorca-Torralba et al., 2022). However, when pain becomes long-term (3–8 weeks depending on the species, strain, and animal model), selective destruction or inactivation of noradrenergic neurons appears not to modify or dampen neuropathic pain (Brightwell and Taylor, 2009; Camarena-Delgado et al., 2021; Llorca-Torralba et al., 2022). Furthermore, the depressive phenotype that usually accompanies long-term neuropathic pain (Yalcin et al., 2014; Barthas et al., 2015; Alba-Delgado et al., 2016) is abolished by intra-LC administration of lidocaine (Camarena-Delgado et al., 2021) and through chemogenetic approaches targeting that structure (Llorca-Torralba et al., 2022), suggesting that LC activation contributes to pain-induced depression. As such, it appears that LC activity can contribute to early analgesia but also to late pronociception and depressive-like behavior (Llorca-Torralba et al., 2022) although the mechanisms underlying this plasticity are still unclear.

Glutamate is the primary excitatory neurotransmitter in the synaptic afferents received by noradrenergic neurons and these inputs arrive from different regions of the CNS, such as the paragigantocellularis (PGi) nucleus and neocortex (Ennis and Aston-Jones, 1988; Singewald and Philippu, 1998). Evidence suggests that altered glutamatergic regulation might be involved in LC plasticity in nerve-injured animals and indeed, short-term peripheral nerve injury (1 week) increases NMDAR1 activity and pCAMKII following noxious stimulus in a model of neuropathic pain (Alba-Delgado et al., 2012b). Furthermore, long-term pain after nerve injury (6 weeks) increases the basal extracellular glutamate concentration in the LC by downregulating glutamate transporter 1 (GLT1) expression, an astroglial glutamate transporter (Kimura et al., 2015). In contrast to glutamate, GABA acts as the principal inhibitory neurotransmitter on LC neurons *via* the GABA-A and GABA-B receptors (Shefner and Osmanovic, 1991; Corteen et al., 2011), and it also inhibits evoked glutamate release *via* presynaptic GABA-B receptors (Suto et al., 2014). LC neurons receive direct GABAergic inputs and it has been speculated that this inhibitory contribution originates from local GABAergic neurons (Aston-Jones et al., 2004; Jin et al., 2016). Furthermore, spinal nerve ligated (SNL) rats show increased basal levels of GABA release and more intense immunoreactivity for the GABA-synthesizing enzyme glutamic acid decarboxylase (GAD) in the LC 1 week after SNL (Yoshizumi et al., 2012). Hence, GABA tone appears to be enhanced after nerve injury.

These data suggest that a dysregulation of glutamatergic and GABAergic neurotransmission is associated with neuropathic pain. However, alterations to the vesicular glutamate transporters 1 and 2 (VGLUT1 and VGLUT2), and to the vesicular inhibitory amino acid transporter (VGAT) at different time points of neuropathy have not yet been examined. These transporters are markers of excitatory and inhibitory synapses, respectively. Indeed, fast glutamate release at the neuronal synapse relies on small vesicles carrying one vesicular glutamate transporter for vesicular glutamate storage (Fremeau et al., 2004a,b). Although the expression of VGLUT1 and 2 overlap in some regions, one transcript usually predominates in given regions (Fremeau et al., 2001; Herzog et al., 2001; Sakata-Haga et al., 2001; Kaneko and Fujiyama, 2002). Thus, in adults, VGLUT1 is mainly expressed by excitatory synapses originating from excitatory neurons in the cerebral cortex, cerebellum, and hippocampus, whereas VGLUT2 is most abundant in those coming from principal neurons in the diencephalon (thalamus, hypothalamus), brainstem, and spinal cord (SC). Similarly, VGAT is a marker for synapses from GABA-releasing neurons. We previously reported stronger expression of tyrosine hydroxylase (TH), pCREB, and c-Fos, as well as increased electrophysiological activity of LC cells in association with long-term pain, coinciding with the onset of anxiodepressive disorders (Alba-Delgado et al., 2013, 2018, 2021; Llorca-Torralba et al., 2019a,b, 2022). Thus, we extended these studies by exploring the expression of markers of excitatory and inhibitory synapses in LC, and also, we evaluated the expression of a marker of apoptosis, cleaved caspase-3 (CC3), because CC3 elevation in the LC and other brain areas has been related to depressive-like behaviors (Gonzalez and Aston-Jones, 2008; Todorovic et al., 2019).

In the light of the above, we have explored the density of puncta expressing excitatory and inhibitory vesicular transporters (VGLUT1, VGLUT2, and VGAT), as well as the marker of apoptosis CC3 in the LC by immunohistochemistry at two different times after neuropathic pain induction. Moreover, ultrastructural features of the noradrenergic LC neurons were evaluated in long-term neuropathic pain animals (CCI28d). In the short-term, 7 days after nerve injury, animals have fully developed sensory hypersensitivity, while after long-term neuropathic pain, 28 days after nerve injury, the pain phenotype is accompanied by anxiety and depressive symptoms.

## Materials and methods

### Animals and experimental design

All the experimental procedures were approved by the Committee for Animal Experimentation at the University of Cadiz, and were carried out in accordance with guidelines for the care and use of laboratory animals: the European Commission directive 2010/63/EU and the Spanish Royal Decree 53/2013. After 1 week of habituation under standard conditions (water and food *ad libitum*, constant room temperature 22 ± 1°C, 12 h light/dark cycle), male Sprague-Dawley rats (250–300 g) were subjected to neuropathic pain for 7 or 28 days. The experimenter was blind to the animal's status in all the behavioral assessments.

### Neuropathic pain: Chronic constriction injury model

Neuropathic pain was induced by chronic constriction injury (CCI) of the common left sciatic nerve (Bennett and Xie, 1988; Bravo et al., 2012). Rats were anesthetized with an intraperitoneal (ip) injection of 100 mg/kg ketamine and 20 mg/kg xylazine, and the common left sciatic nerve was exposed at the mid-thigh level proximal to the sciatic trifurcation and separated from the adjacent tissue. Four loose ligatures were tied around the dissected nerve using chromic catgut sutures (4–0) with a 1.5 mm interval between each pair of ligatures. The overlying muscle was closed in layers with synthetic absorbable surgical suture (4–0) and the skin was sutured with silk thread (2–0). In Sham-operated rats, an identical dissection was performed but the sciatic nerve was not ligated. The animals were analyzed 7 (short-term, CCI7d) or 28 days (long-term, CCI28d) post-surgery.

### Sensory pain behavior

#### Mechanical hypersensitivity (von Frey test)

In order to test whether animals developed neuropathy, at the end of the experiment, mechanical allodynia was measured in rats using an automatic von Frey apparatus (Dynamic Plantar Anesthesiometer Cat. No. 37400-002, Ugo Basile, Italy; Bravo et al., 2013). Animals were randomly placed in plastic cages with an operable metal grid to which they had been habituated for 30 min prior to the test. A vertical force was applied to the hind paw that increased from 0 to 50 g over a period of 20 s, and the threshold was determined as the average of two values that induced a withdrawal response (with a 50 g cut-off).

#### Cold hypersensitivity (acetone test)

Animals were placed individually into Plexiglas chambers on a metal grid, and a drop of acetone (100 μl) was applied to the center of the ipsilateral and contralateral hind paw with a pipette (Bravo et al., 2012). The acetone was applied four times to each hind paw Alternately at 5 min intervals and the responses were recorded over 1 min according to the following scale: 0, no response; 1, quick withdrawal, flick or stamping of the paw; 2, prolonged withdrawal or repeated flicking of the paw; and 3, repeated flicking of the paw with persistent licking directed at the ventral side of the paw. The cumulative score for each rat was obtained by summing the score and dividing it by the number of assays.

### Emotional-like behavior

#### Elevated zero maze test

Anxiety-like behavior was evaluated in the elevated zero maze (EZM) test (Llorca-Torralba et al., 2018). Animals were placed in a black, 10 cm wide circular track (120 cm in diameter), elevated 70 cm above the ground, with two opposing enclosed arms and two open arms, and with a 7-mm-high edge to prevent falls. The time spent in the open arm (%, a measure of anxiety) was monitored over a 5-min test period using the SMART video software (Spontaneous Motor Activity Recording and Tracking: Panlab, S.L., Barcelona, Spain). Anxiety-like behavior was defined as a decrease in the time spent in the open arm.

#### Forced swimming test

Depressive-like behavior was evaluated in the forced swimming test (FST) (Llorca-Torralba et al., 2019a). Animals were placed individually in large plastic cylinders filled to a depth of 30 cm with water at 25 ± 1°C, over two different sessions: a 15-min pre-test and a 5-min test performed 24 h later. The predominant behaviors were recorded (climbing, swimming, or immobility) and scored in each 5 s period of the 300 s test session using customized software (Red-Mice, Cadiz, Spain). Immobility behavior was determined when no activity was observed other than the movements necessary to keep the animal's head above water. Climbing behavior was measured when the rats made vigorous upward movements with their forepaws in and out of the water. Swimming was considered the predominant behavior when the rats moved around the cylinder. Depressive-like behavior was defined as an increase in mean immobility.

### Perfusion, microtomy, and immunohistochemistry

The distribution of VGLUT1 and VGLUT2 in the LC region was assessed in brain tissue from the rats. Animals were deeply anesthetized with sodium pentobarbital and perfused transcardially with 4% paraformaldehyde (PFA) in phosphate buffer (PB, 0.1 M) for confocal microscopy (*n* = 5–6) studies and with an additional 0.5% glutaraldehyde for electron microscopy (EM) studies (*n* = 3). The rat's brain was removed and cryoprotected in 30% sucrose in PB (0.1 M) for 48 h, and coronal vibratome sections (50 μm thick: Leica VT 1000E) of the region containing the LC were collected in four series and processed “free-floating” for immunohistochemistry. Briefly, sections were washed in phosphate-buffered saline (PBS) and then incubated for 1 h in 10% normal donkey serum (NDS: Abcys) in PBS with 0.3% Triton X-100 (PBST: Sigma-Aldrich). The sections were then incubated for 48 h at 4°C with the appropriate primary antibody or antibody cocktail (see Table 1) diluted in PBST with 0.5% of NDS as follows: (a) triple immunostaining using antibodies against TH, VGLUT2, and VGAT; (b) double staining with antibodies against TH and VGLUT1; (c) double staining using antibodies against dopamine beta hydroxylase (DBH) and CC3. In order to detect the expression of CC3 in GFAP^+^ or NeuN^+^ cells, the brains of the second group of animals (*n* = 2–3 per group) were processed and 30-μm-thick coronal sections were triple stained using antibodies against NeuN, CC3, and GFAP, or against DBH, CC3, and GFAP.

**Table 1:** List of primary and secondary antibodies used in the study.

	**Dilution**	**Company**
**Primary antibodies**
Guinea pig anti-VGLUT1 (*Vesicular Glutamate Transporter 1*)	1:2,000	Millipore, AB5905
Guinea pig anti-VGLUT2 (*Glutamate Transporter 2*)	1:2,000	Millipore, AB2251-I
Rabbit anti-VGAT (*Vesicular GABA Transporter*)	1:1,000	Synaptic systems, 131 002
Mouse anti-DBH (*Dopamine Beta Hydroxylase*)	1:1,000	Millipore, MAB308
Rabbit anti-CC3 (*Cleaved Caspase-3* (*Asp175*))	1:500	Cell signaling, #9661S
Goat anti-GFAP (*Glial fibrillary acidic protein*)	1:1,000	Abcam Ab53554
Rabbit anti-TH (Tyrosine hydroxylase)	1:1,000	Millipore, AB152
Mouse anti-NeuN	1:500	Millipore MAB377
**Secondary antibodies**
Goat anti-Guinea Pig A-647	1:1,000	Invitrogen A-21450
Donkey anti-mouse Alexa-555	1:1,000	Invitrogen A31570
Biotinylated Donkey anti-guinea pig	1:200	Jackson 706-065-148
Biotinylated Donkey anti-goat	1:200	Jackson 705-065-147
Donkey anti-rabbit Alexa-488	1:1,000	Invitrogen A21206
Streptavidin Alexa 568 conjugate	1:1,000	Invitrogen S11226
Donkey anti-mouse Alexa-647	1:1,000	Invitrogen A31571

After washing, the sections were incubated for 1 h at room temperature (RT) with secondary antibodies conjugated to the appropriate fluorochrome or streptavidin (see Table 1), also diluted in PBST. Finally, sections were washed in PB (0.1 M), mounted on slides, and coverslipped using fluorescence mounting medium (Dako).

### Quantification of VGLUT1, VLGUT2, and VGAT puncta in the LC

VGLUT1 was distributed around the pericoerulear region but not specifically surrounding the soma of TH^+^ cells (**Figure 2**; Barr and Van Bockstaele, 2005). Consequently, the density of VGLUT1 puncta was evaluated in the entire LC region. Confocal z-stacks of three to four quadrants in a total of three LC sections per rat were acquired with a 1 μm step size and with a 63× oil immersion objective on a Zeiss Axio Observer Z1 Confocal microscope. The images were analyzed using a Macro generated from ImageJ software that converted the image to RGB color. The density of the VGLUT1 puncta was quantified using a threshold signal intensity (170 on a 0–255 Brightness scale) that maximized the selection of puncta and minimized the background noise. The results were expressed as the number of puncta/μm^2^.

VGLUT2 and VGAT were expressed around the pericoerulear region and specifically, surrounding the somata of TH^+^ cells. Thus, the density of puncta expressing VGLUT2 or VGAT puncta was evaluated in the entire LC region but also, surrounding noradrenergic somata, using a protocol described previously (Guirado et al., 2018) and adapted to the LC. Confocal z-stack of three to four quadrants in a total of three LC sections per rat were acquired with 1 μm step size with a 63× oil immersion objective by using a confocal microscope (FV 10i; Olympus, Japan). For perisomatic study of VGLUT2 and VGAT markers, a mean of 30 to 35 neurons was randomly selected in the sections acquired. The profile of the noradrenergic soma of these neurons was drawn manually to assess the density of the puncta around the perimeter. This manual selection was enlarged by 0.5 μm in order to define the perisomatic area. The linear density of VGLUT2 and VGAT immunoreactive puncta within an optical section was analyzed manually using ImageJ (National Institutes of Health, Bethesda, Maryland). The results were expressed as the number of puncta/μm^2^.

The expression of all the vesicular transporter markers studied was represented along the dorsal–ventral axis of the LC (−9.72 to −9.96 from Bregma, three slices per animal; Llorca-Torralba et al., 2022).

### Quantification of the soma size of noradrenergic LC neurons

To detect changes in soma size, the mean soma area per group was calculated in 30–35 randomly selected TH^+^ cells per animal. Each TH^+^ soma was outlined manually using a computer mouse and the cross-sectional area was calculated with ImageJ software. The results were represented in μm^2^.

### Electron microscopy

For the EM studies, Sprague-Dawley rats (Sham and CCI28d, *n* = 3) were perfused transcardially with fixative containing 4% formaldehyde and 0.5% glutaraldehyde in PB (0.1 M, pH 7.4). Coronal vibratome sections (50 μm thick) through the rostrocaudal extent of the LC were collected in PB (0.1 M) and left for 20 min in 1% sodium borohydride in PB (0.1 M) to remove reactive aldehydes. After rinsing extensively in PB (0.1 M), the sections were then incubated with the primary antibody. TH was detected using the avidin–biotin–peroxidase (ABC) method. The sections were first blocked with 10% NDS in PB (0.1 M) for 45 min at RT and then probed for 48 h at 4°C with a rabbit antiserum against TH (Millipore) diluted 1:1,000 in PB containing 1% NDS and 0.05% sodium azide. A biotinylated donkey anti-rabbit (Thermo Scientific, Fremont, CA, USA) antibody, diluted 1:200 in PB, was used to detect the antibody binding for 2 h at RT, and the sections were then incubated for 2 h at RT with an avidin-biotinylated horseradish peroxidase complex (ABC: Vector Labs. Burlingame, CA, USA) diluted 1:200 in PB. The peroxidase reaction was then developed over 5 min at RT using 0.05% 3,3-diaminobenzidine tetrahydrochloride (DAB: Sigma-Aldrich, St. Louis, MO, USA) and 0.003% hydrogen peroxide in PB. Sections were carefully rinsed in PB after each step.

After performing immunohistochemistry, the sections were treated for 45 min at RT with 1% osmium tetroxide (Electron Microscopy Sciences, Hatfield, PA, USA) containing 7% glucose in PB. Subsequently, the sections destined for EM were stained with uranyl acetate (Electron Microscopy Sciences) in maleate buffer (pH 4.5), dehydrated through a graded ethanol series and in propylene oxide (Fluka AG, Buch, Switzerland), and flat-embedded in Durcupan (Fluka AG) between slides and coverslips. Durcupan was polymerized overnight at 60°C and the flat-embedded sections were examined under a light microscope to select the LC tissue containing TH^+^ neurons for further analyses. The selected material was re-embedded in Durcupan and serial ultrathin sections (60 nm thick) were obtained in an ultramicrotome and mounted on formvar-coated nickel grids. The grids were contrasted with 1% uranyl acetate in 70% ethanol for 1 min and then in a 0.2% lead citrate contrasting solution for 5 min (Venable and Coggeshall, 1965). The sections were observed under a transmission electron microscope (Jeol JEM 1010) at 100 kV.

Ultrathin sections of tissue immediately adjacent to the fourth ventricle in the region of the LC were captured by EM at 4,000× magnification. The densities of mitochondria and lysosomes found in a mean of 9 to 10 neurons per animal (*n* = 3 per group) were analyzed and the results were represented in μm^2^.

Axons were classified according to the following myelin compaction code (Savigni et al., 2013): (A) normally myelinated, myelin in thick, high-electron density, with no signs of decompaction; (B) marginally decompacted myelin, with axons presenting small signs of decompaction in ≤20% of the circumference of the myelin sheath; (C) partially decompacted myelin, with ≥20% of the myelin sheath showing signs of decompaction; (D) fully decompacted myelin with the entire axon ensheathed with multiple layers of thin, moderate-electron dense myelin; and (E) unmyelinated.

### Quantification of CC3 and GFAP in the LC

Confocal z-stack of three to four quadrants in a total of three LC sections per rat were acquired with a 63× oil immersion objective (*n* = 4–5 rats per group) on a Zeiss LSM 880 Confocal microscope with FAST Airyscan (Carl Zeiss Microscopy GmbH, Germany). CC3^+^ cells were quantified manually in the LC region using the ImageJ Cell Counter plugin (National Institutes of Health, Bethesda, Maryland). The results were expressed as the mean number of CC3^+^ cells per animal in the whole LC region and along the dorso-ventral LC axis. The relative GFAP^+^ area was quantified using a threshold (14 on a 0–255 greyscale) for the signal intensity that maximized the selection of expressing cells while minimizing the background noise. A mean of three to four quadrants per section was represented and expressed as the percentage area occupied by GFAP.

### Statistical analysis

All the data were analyzed using Graph-Pad Prism 9.1 (GraphPad San Diego, CA) and Statistic 10.0 software (Statistic, Tulsa, Oklahoma), and they were presented as the mean ± SEM. One-way analysis of variance (ANOVA) was followed by the appropriate post-hoc tests (Tukey test) and an unpaired Student's t-test was used to compare the values between the two groups. In all cases, *p* < 0.05 was considered significant.

## Results

### Neuropathic pain induces nociceptive responses and anxiodepressive-like behavior

**Figure 1:**
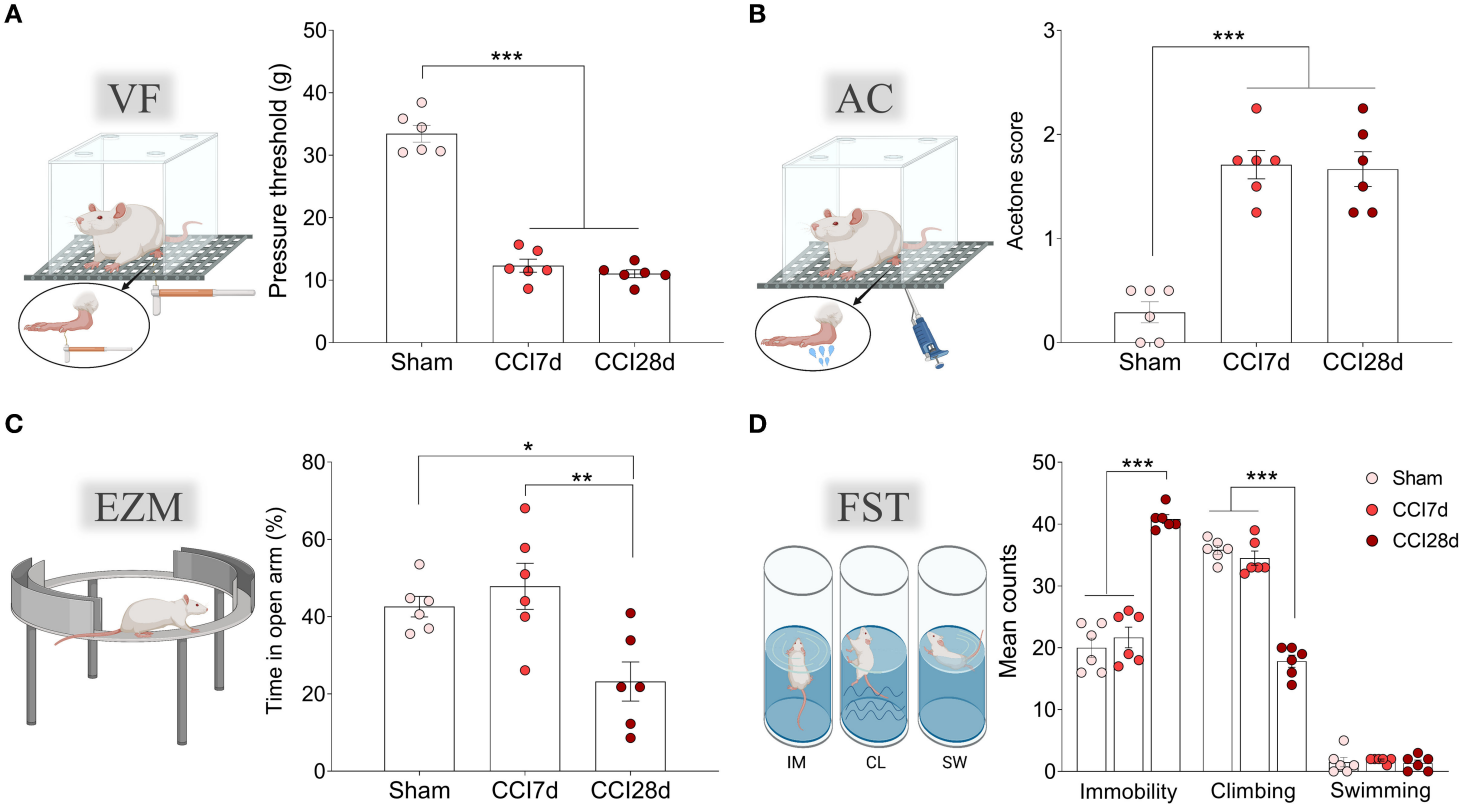
Nociceptive and emotional behaviors after chronic constriction injury. **(A)** Mechanical hypersensitivity (g) of the ipsilateral paw in response to von Frey (VF) hair stimulation (0–50 g, 20 s). **(B)** Thermal hypersensitivity of the ipsilateral paw in response to acetone (AC) application (100 μl). **(C)** Anxiety-related behavior in the elevated zero maze test (EZM) and the relative time spent in the open areas (5 min test). **(D)** Depressive-related behavior in the forced swimming test (FST) relative to the predominant behavior (IM, immobility; CL, climbing; SW, swimming: 5 min test). Each bar represents the mean ± SEM of six animals/group: *p < 0.05, **p < 0.01, ***p < 0.001 as assessed by one-way ANOVA followed by Tukey's post-test.

As expected, nerve-injured animals displayed pain hypersensitivity in response to mechanical and thermal stimuli, both at 7- and 28-days post-surgery (*p* < 0.001: Figures 1A,B). No differences were observed in the contralateral paw between the groups when evaluated by the von Frey test (values obtained: Sham 34.79 ± 3.91, CCI7d 38. 57 ± 7.16, CCI28d 39.28 ± 7.10) or acetone test (values obtained: Sham 0.13 ± 0.21, CCI7d 0.25 ± 0.22, CCI28d 0.21 ± 0.25). However, when anxiety and depressive-like behaviors were evaluated (Figures 1C,D), CCI28d animals spent significantly less time in the open arms of the EZM than Sham (*p* < 0.05) and CCI7d (*p* < 0.01) animals, reflecting an anxiogenic state (Figure 1C). In the FST, the CCI28d rats spent more time in the immobile state relative to the Sham and CCI-7d animals (*p* < 0.001), whereas their climbing time decreased (*p* < 0.001), indicative of a depressive-like state. No changes were observed in swimming behavior (Figure 1D).

### Expression of VGLUT1, VGLUT2, and VGAT in the LC

**Figure 2:**
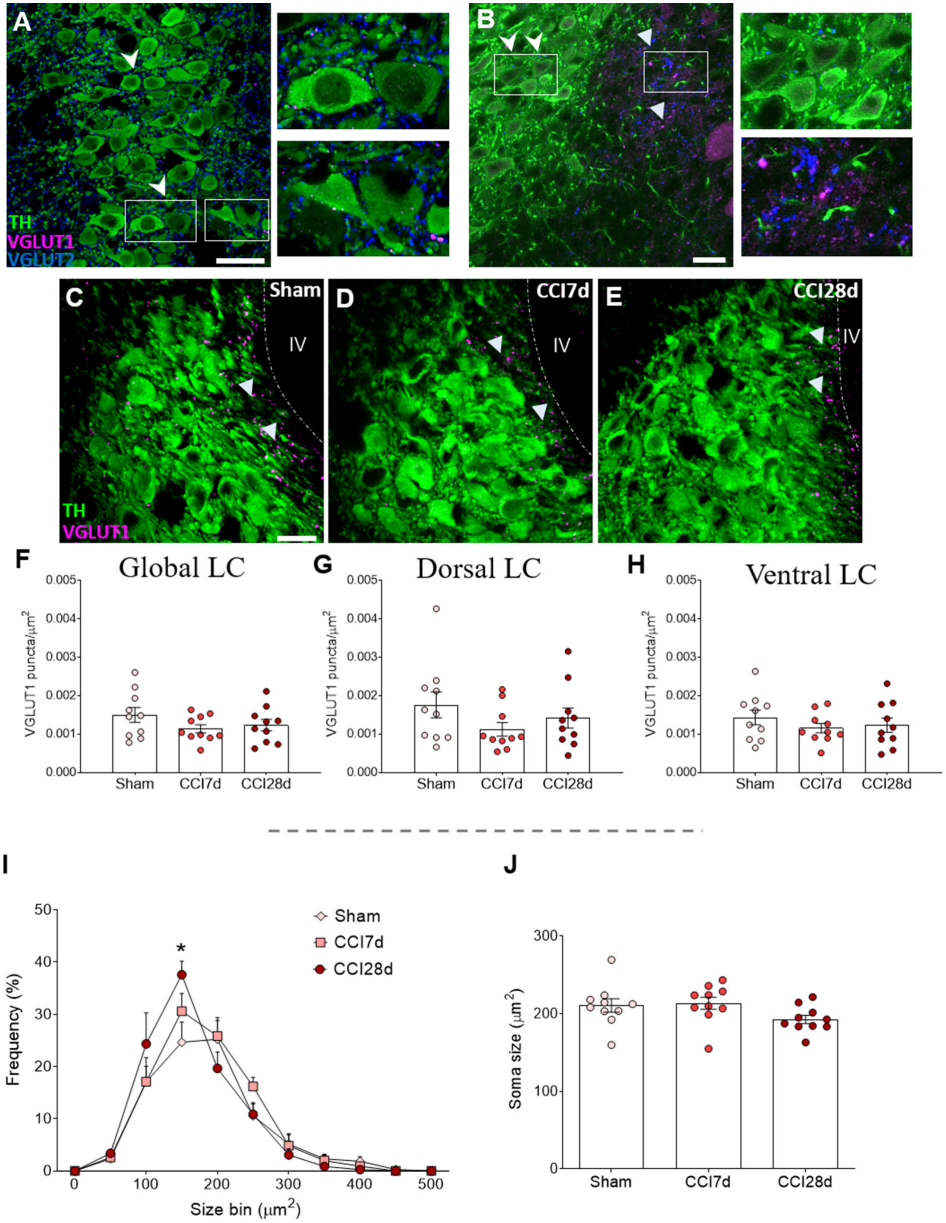
Study of VGLUT1 expression in the LC of neuropathic pain rats. **(A,B)** Localization of the vesicular glutamate transporter 1 (VGLUT1) and vesicular transporter 2 (VGLUT2) in the LC region. The arrowheads point to VGLUT2^+^ puncta in both the pericoerulear and perisomatic area (TH green; VGLUT1 magenta; VGLUT2 blue: scale bar, 20 μm). The triangles point to VGLUT1^+^ puncta mainly in the pericoerulear area. **(C–E)** Representative confocal images of the LC showing neurons expressing TH and puncta-expressing VGLUT1 in **(C)** Sham, **(D)** CCI7d, and **(E)** CCI28d rats (TH green; VGLUT1 magenta: scale bar, 20 μm). **(F–H)** Quantification of the density of VGLUT1^+^ puncta in the **(F)** global, **(G)** dorsal, and **(H)** ventral LC. The data represent the mean ± SEM of the number of the puncta/μm^2^ (obtained from three to four quadrants in a total of three LC sections per rat). The graphs depict **(I)** the frequency distribution of the TH^+^ soma size and **(J)** the mean of soma size for each experimental group. The data are presented as the mean ± SEM of 30–35 random neurons per animal: *p < 0.05 vs. Sham, one-way ANOVA followed by a Tukey's post-test.

As a first approach, we assessed the distribution of excitatory VGLUT1 and VGLUT2 in the entire LC region. In accordance with previous studies, VGLUT1 expression was clearly evident around the pericoerulear region while VGLUT2 expression was prominent in both the pericoerulear region and surrounding the somata (Figures 2A,B: Barr and Van Bockstaele, 2005). When the density of VGLUT1 puncta was quantified across the whole LC region (Figures 2C–E), no significant differences were detected between the three experimental groups (Figures 2F–H). Similarly, there were no significant differences between the different groups in the size of the neuronal somata when the area they occupied was explored (Figures 2I,J). However, the frequency distribution of the soma sizes revealed an increase in the proportion of intermediate cells following CCI, which was significantly different in the CCI28d rats compared to the Sham group (150 μm^2^, *p* < 0.05; Figure 2I).

**Figure 3:**
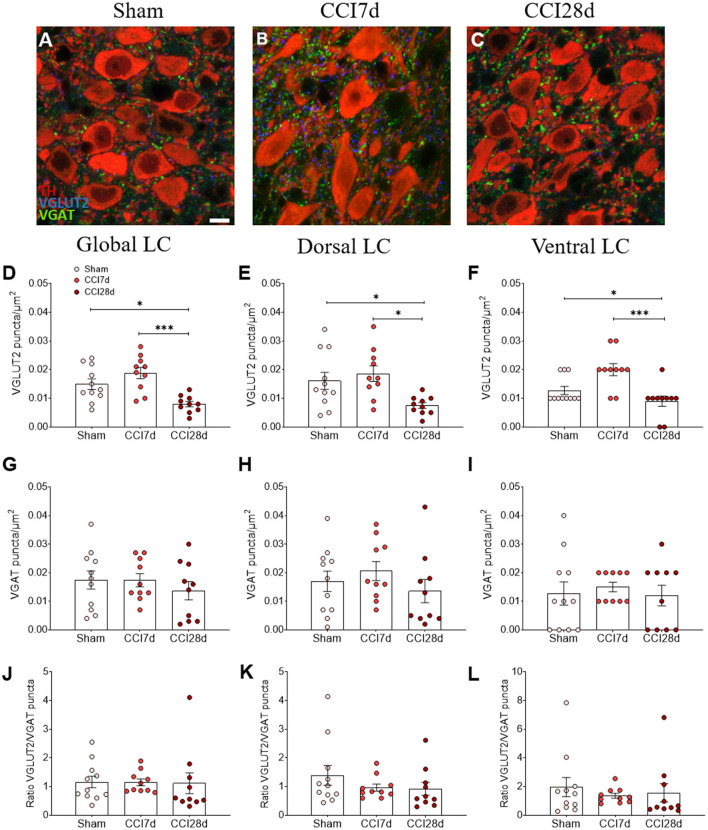
VGLUT2 and VGAT expression in the LC of neuropathic pain rats. **(A–C)** Representative confocal images showing the density of puncta expressing VGLUT2 and VGAT in the LC of **(A)** Sham, **(B)** CCI7d, and **(C)** CCI28d rats (TH red; VGLUT2 blue; VGAT green: scale bar, 10 μm). **(D–F)** Quantification of the density of puncta expressing VGLUT2 in the **(D)** global, **(E)** dorsal, and **(F)** ventral LC. **(G–I)** Quantification of the density of VGAT puncta in the **(G)** global, **(H)** dorsal, and **(I)** ventral LC. **(J–L)** Representation of the ratio of VGLUT2/VGAT puncta in the **(J)** global, **(K)** dorsal, and **(L)** ventral LC. The data represent the mean ± SEM of the number of the puncta/μm^2^ (obtained from three to four quadrants in a total of three LC sections per rat): *p < 0.05, ***p < 0.001 vs. the corresponding group, one-way ANOVA followed by a Tukey's post-test.

In terms of the density of VGLUT2 or VGAT immunoreactive puncta in the LC region (Figure 3), one-way ANOVA revealed a significant effect of CCI surgery on these parameters (*p* < 0.001), an effect that persisted in the dorsal (*p* < 0.01) and ventral region of the LC (*p* < 0.001). Indeed, there was a significant decrease in VGLUT2^+^ puncta in the LC of CCI28d rats relative to the Sham (*p* < 0.05) and CCI7d animals (*p* < 0.001; Figure 3D), an effect that was particularly evident in the ventral region of the LC (Figures 3E,F). However, the density of VGAT puncta or the VGLUT2/VGAT ratio was no different between the groups of animals.

**Figure 4:**
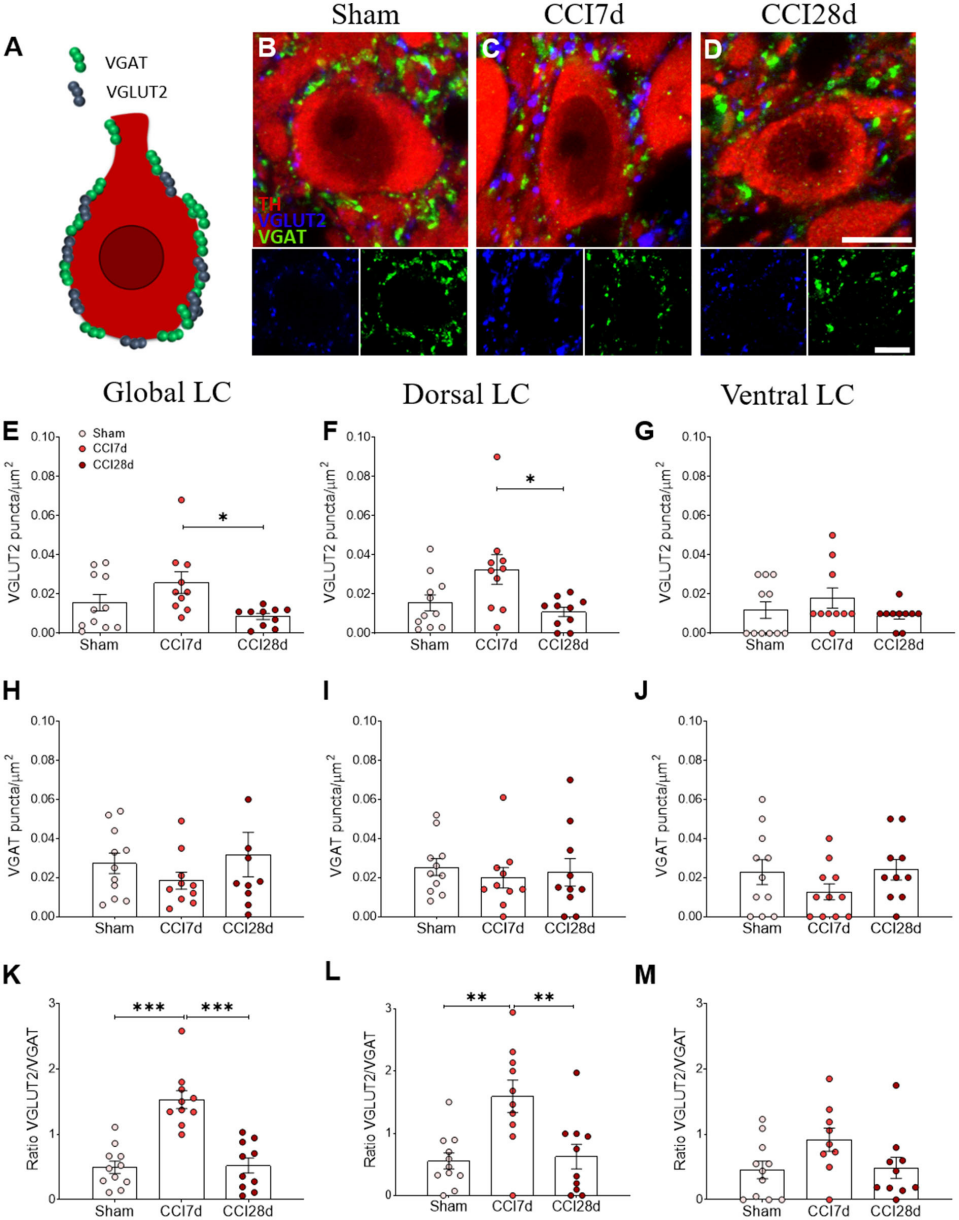
Perisomatic puncta expressing VGLUT2 and VGAT in LC neurons of neuropathic pain rats. **(A–D)** Representative confocal images showing perisomatic density of puncta expressing VGLUT2 and VGAT in the LC of **(B)** Sham, **(C)** CCI7d, and **(D)** CCI28d rats (TH red; VGLUT2 blue; VGAT green: scale bar, 10 μm). **(E–G)** Quantification of the VGLUT2 expression in the **(E)** global, **(F)** dorsal, and **(G)** ventral LC. **(H–J)** Quantification of VGAT expression in the **(H)** global, **(I)** dorsal, and **(J)** ventral LC. **(K–M)** Representation of the ratio of VGLUT2/VGAT puncta in the **(K)** global, **(L)** dorsal, and **(M)** ventral LC. The data are presented as the mean ± SEM of the number of the puncta/μm^2^ (obtained from 30 to 35 neurons from three LC sections per rat): *p < 0.05, **p < 0.01, ***p < 0.001 vs. the corresponding group, one-way ANOVA followed by a Tukey's post-test.

In addition, the density of VGLUT2 and VGAT puncta surrounding the soma of noradrenergic neurons was analyzed (Figure 4) and one-way ANOVA of the VGLUT2^+^ puncta revealed a significant effect of CCI surgery (*p* < 0.05) in the dorsal (*p* < 0.05) but not the ventral region of the LC (Figures 4F,G). Indeed, an overall increase of VGLUT2^+^ puncta was evident in the LC of CCI7d rats relative to the CCI28d animals (*p* < 0.05: Figure 4E), an effect that was detected in the dorsal but not in the ventral region of the LC (*p* <0.05: Figures 4F,G). The density of VGAT immunoreactive puncta was not altered in any group, although there was a prominent increase in the VGLUT2/VGAT ratio in CCI7d rats relative to the Sham and CCI28d animals (*p* < 0.01: Figure 4K). This increase in the VGLUT2/VGAT ratio was notable in the dorsal (*p* <0.01: Figure 4L) but not the ventral LC (Figure 4M).

### Ultrastructural changes of noradrenergic LC neurons

**Figure 5:**
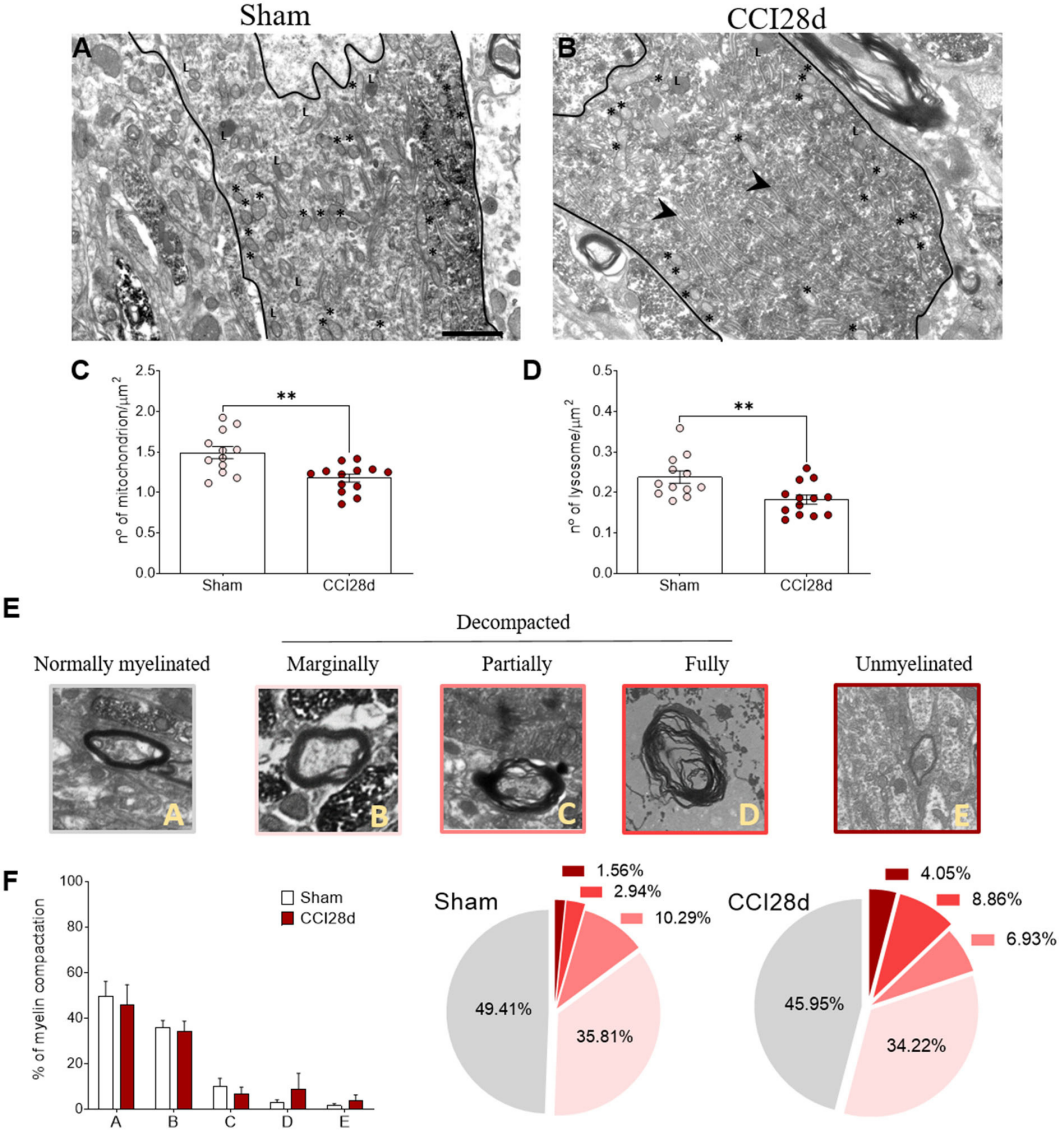
Ultrastructural studies of noradrenergic LC neurons in neuropathic pain rats. Representative electron microscopy images of **(A)** Sham and **(B)** CCI28d rats. The asterisks indicate mitochondria and the arrowheads point to the dilated ER: L, lysosome. Scale bar: 2 μm. **(C,D)** Quantification of the number of **(C)** mitochondria and **(D)** lysosomes. **(E,F)** Representative images and quantification of the relative myelin compaction scored from A to E as axons with normal myelin **(A)**, marginally **(B)**, partially **(C)**, fully decompacted **(D)**, and unmyelinated **(E)**.

In a different set of animals, the ultrastructural features of the noradrenergic LC neurons of Sham and CCI28d animals were explored (Figures 5A,B). The EM images from Sham animals were similar to those described previously (Groves and Wilson, 1980), whereas long-term nerve injury (CCI28d) appeared to induce changes in LC neurons evidenced mainly through the presence of more dilated ER and mitochondria in EM images. Mitochondria and lysosomes were quantified in a mean of nine to ten neurons per animal (*n* = 3 per group), revealing a significant decrease in their number/μm^2^ (^**^p < 0.01: Figures 5C,D). When myelin compaction was assessed, the percentage of (A) normally myelinated, (B) marginally decompacted myelin, and (C) partially decompacted myelin was similar in Sham and CCI28d rats. However, there was a trend in the CCI28d group toward an increase in the level of (D) fully decompacted myelin (CCI28d 8.96%, Sham 2.94%) and of (E) unmyelinated axons (CCI28d 4.05%, Sham 1.56%: Figures 5E,F).

### Expression of CC3 in DBH^+^ and DBH^–^ LC cells

**Figure 6:**
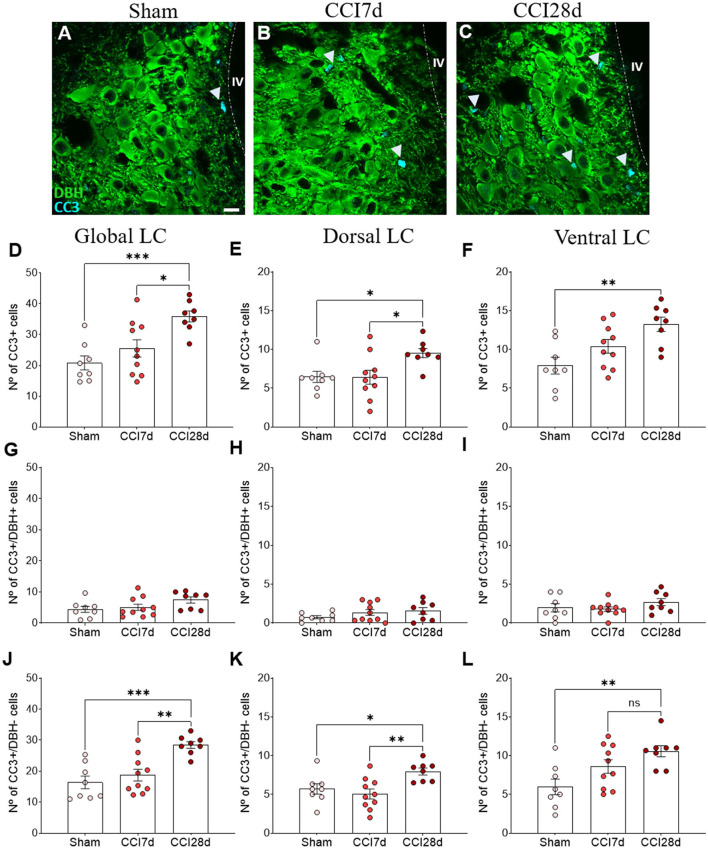
Cell damage in the LC of neuropathic pain rats. **(A–C)** Representative confocal images of CC3 expression in DBH^+^ neurons of the LC in **(A)** Sham, **(B)** CCI7d, and **(C)** CCI28d rats (DBH green, CC3 blue: scale bar, 10 μm). **(D–F)** Quantification of the number of CC3^+^ cells in the **(D)** global, **(E)** dorsal, and **(F)** ventral LC. **(G–I)** Quantification of the number of CC3^+^/DBH^+^ cells in the **(G)** global, **(H)** dorsal, and **(I)** ventral LC. **(J–L)** Quantification of the number of CC3^+^/DBH^−^ cells in the **(J)** global, **(K)** dorsal, and **(L)** ventral LC. The data are presented as the mean ± SEM of the number of CC3^+^ cells (obtained from three to four quadrants in a total of three LC sections per rat): *p < 0.05, **p < 0.01, ***p < 0.001 vs. the indicated group, one-way ANOVA followed by a Tukey's post-test.

In order to determine the cell damage provoked in the LC as a result of CCI, we examined the density of CC3^+^ cells in this structure (Figures 6A–C). One-way ANOVA revealed a significant effect of CCI surgery on the global LC (*p* < 0.001), which was also evident when the dorsal (*p* < 0.05) and ventral regions of the LC (*p* < 0.01) were analyzed separately. Tukey's multiple comparisons test revealed a significant increase in the density of CC3^+^ cells in CCI28d rats relative to the Sham (*p* < 0.001) and CCI7d animals (*p* < 0.05) when the LC is considered globally (Figure 6D). This was also the case in the dorsal region of the LC (Figure 6E), whereas in the ventral LC domain, there was an increase in the number of CC3^+^ cells in CCI28d rats relative to Sham animals (*p* < 0.01), yet not when compared to the LC of CCI7d rats (Figure 6F).

In the images captured, most CC3^+^ cells did not express DBH, suggesting that another type of cell was expressing CC3. When the density of CC3^+^ in DBH^+^ and DBH^−^ was considered, the low density of CC3^+^ in DBH^+^ cells was similar in each experimental group (Sham 4.4 ± 2.7, CCI7d 5.1 ± 3.1, CCI28d 7.43± 2.7: Figures 6G–I).

However, in the DBH^−^ cells, one-way ANOVA revealed a significant effect of CCI surgery in the global (*p* < 0.001) and as well as in the dorsal and ventral region (*p* < 0.01) of LC. Tukey's multiple comparisons tests revealed that CC3 was detected more often in DBH^−^ cells at CCI28d than in the Sham rats (*p* < 0.001) and this difference was lesser extent compared to CCI7d rats (*p* < 0.01: Figure 6J). The differences between the Sham and CCI28d were also evident in the dorsal but not in the ventral region of the LC (*p* < 0.01: Figures 6K,L).

### Expression of CC3 in GFAP^+^ LC cells

**Figure 7:**
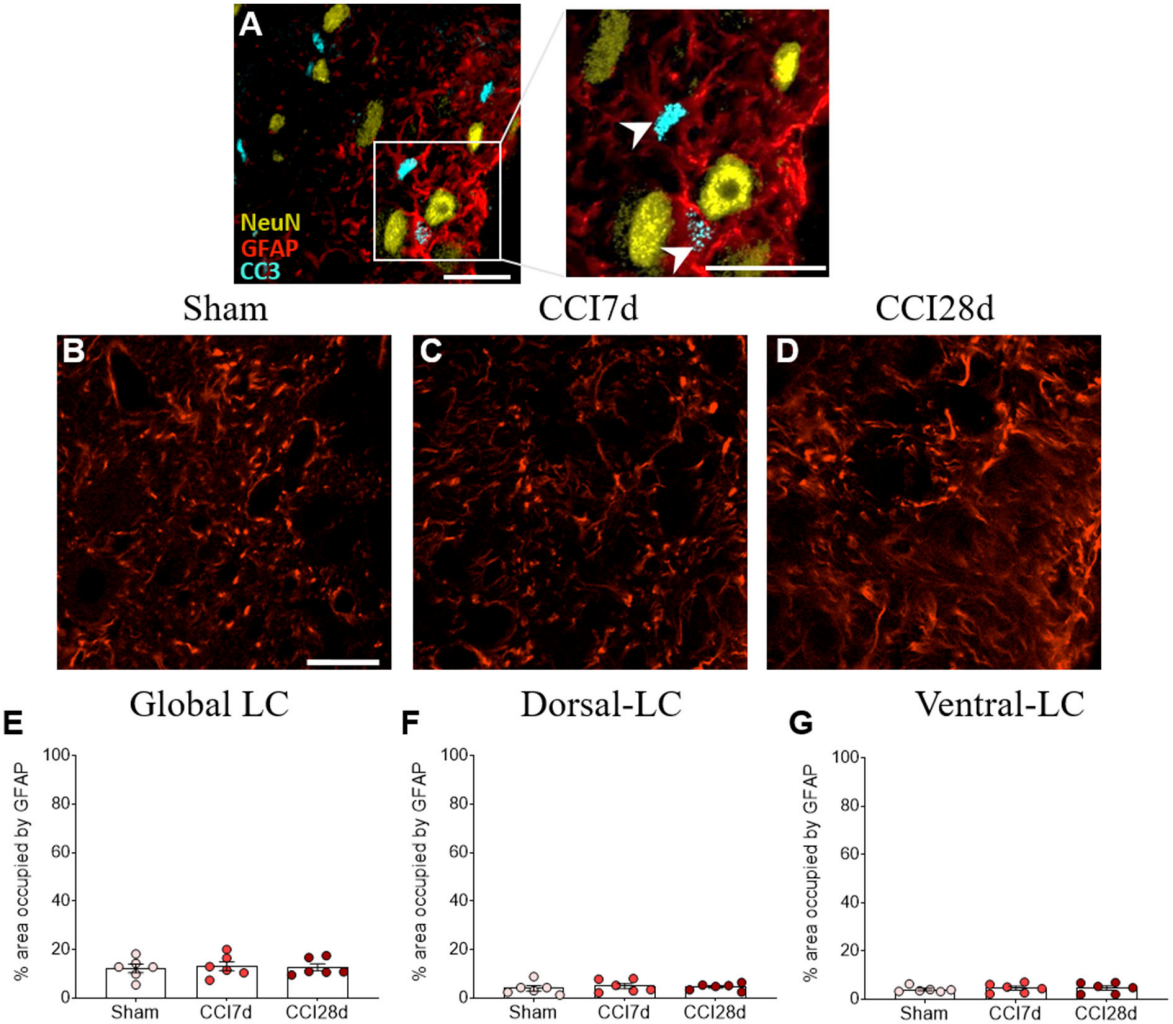
Study of the CC3 in GFAP^+^ or NeuN^+^ cells in the LC. **(A)** Representative confocal images of CC3^**+**^/GFAP^**+**^ cells without NeuN (NeuN yellow, GFAP red, CC3 blue; scale bar, 20 μm). **(B–D)** Representative confocal images of GFAP expression in **(B)** Sham, **(C)** CCI7d, and **(D)** CCI28d rats. **(E–G)** Quantification of the area occupied by GFAP in the **(E)** global, **(F)** dorsal, and **(G)** ventral LC. The data are presented as the mean ± SEM of three to four quadrants per section (three slices), one-way ANOVA followed by a Tukey's post-test.

**Figure 8:**
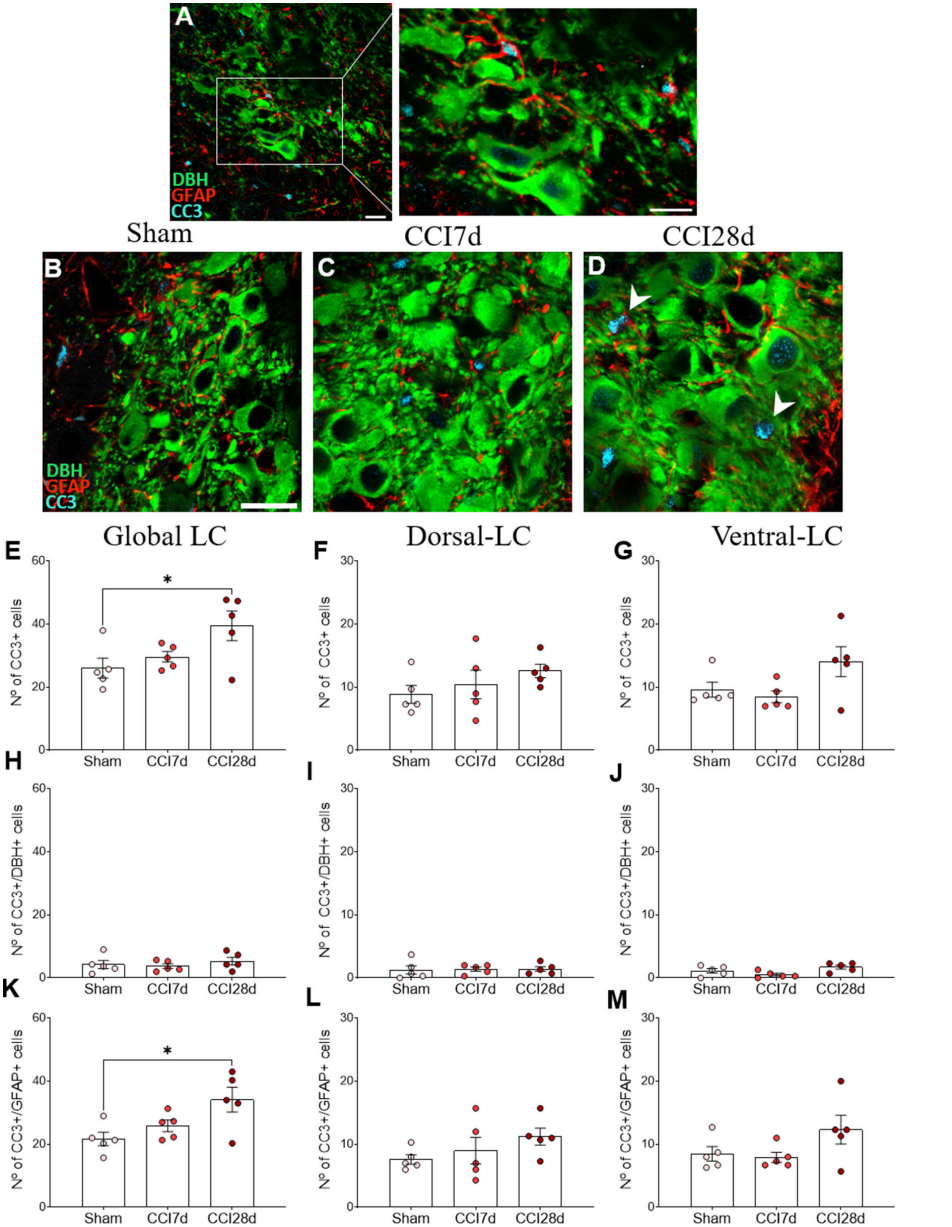
CC3 expression in DBH^+^ and GFAP^+^ LC cells of neuropathic pain rats. **(A)** Representative confocal images of CC3 expression in DBH^+^ neurons in the LC of **(B)** Sham, **(C)** CCI7d, and **(D)** CCI28d rats (DBH green; GFAP red; CC3 blue: scale bar, 20 μm). **(E–G)** Quantification of the number of CC3^+^ cells in the **(E)** global, **(F)** dorsal, and **(G)** ventral LC. **(H–J)** Quantification of the number of CC3^+^/DBH^+^ cells in the **(H)** global, **(I)** dorsal, and **(J)** ventral LC. **(K–M)** Quantification of the number of CC3^+^/GFAP^+^ cells in the **(K)** global, **(L)** dorsal, and **(M)** ventral LC. The data are presented as the mean ± SEM of the number of the CC3^+^ cells/μm^2^ obtained from three to four quadrants per section (three slices). *p < 0.05, **p < 0.01, ***p < 0.001 vs. the group indicated, one-way ANOVA followed by a Tukey's post-test.

We performed triple immunofluorescence to determine the identity of the CC3^+^ cells, first combining staining for CC3 with NeuN and GFAP (Figure 7). There was robust co-expression of CC3 with GFAP but not with NeuN in the cells (Figure 7A), suggesting that CC3 was mainly expressed by astrocytes in the LC. As a result, we assessed the co-localization of CC3, DBH, and GFAP (Figures 8A–D), recording the mean number of CC3^+^ cells, and the number of cells immunostained for both CC3 and DBH or GFAP in both entire LC and the dorsal/ventral regions (Figures 8E–M). One-way ANOVA revealed a significant effect of CCI surgery on the expression of CC3 in the LC as a whole (*p* < 0.05), although this was not evident when the dorsal or ventral LC were considered separately. A Tukey's multiple comparisons test revealed that there were significantly more CC3^+^ cells in the CCI28d LC than that of the Sham rats (*p* < 0.05), yet not relative to the CCI7d animals (Figure 8E). There were no differences in the proportion of DBH^+^ cells expressing CC3 between the groups (Figures 8H–J), although as seen previously, the CC3^+^ cells observed in the CCI28d LC coincided with GFAP^+^ cells and again there were differences compared to the Sham group (*p* < 0.05, Tukey's test: Figure 8K). However, this effect was no longer evident when the dorsal or ventral LC regions were considered separately (Figures 8L,M). Although CC3 was co-expressed with GFAP, the quantification of GFAP expression in the LC was similar in all three experimental groups (Figures 7E–G).

## Discussion

This study shows that short-term peripheral nerve injury (CCI7d) increases the expression of markers of excitatory synapses in perisomatic puncta of noradrenergic cells in the LC relative to long-term pain (CCI28d). In addition, long-term pain is associated with a decrease in the density of mitochondria and lysosomes, and an increase of the CC3 marker in glia but not in noradrenergic cells.

The density of VGLUTs and VGAT in the LC was explored here. Vesicular glutamate or GABA transporters are present in glutamatergic or GABAergic vesicles where they serve to transport the corresponding neurotransmitter into the synapse to ensure the release of glutamate or GABA into the synaptic cleft (Roth and Draguhn, 2012; Siksou et al., 2013). Short-term pain (CCI7d) did not produce significant differences in VGLUT1, VGLUT2, or VGAT compared to Sham animals in the region containing the entire LC. However, when perisomatic puncta expressing excitatory (VGLUT2) or inhibitory (VGAT) markers on noradrenergic cells were considered, an increase in VGLUT2 and a tendency to decrease the expression of VGAT was seen at CCI7d relative to CCI28d animals. The increase in the VGLUT2/VGAT ratio in CCI7d rats relative to Sham and CCI28d animals suggests an increase in the excitatory/inhibitory balance of neurotransmission at this time point of nerve injury. Whereas VGLUT1 is localized in excitatory neurons in the cerebral cortex, VGLUT2 is found mostly in synapses from neurons in the diencephalon (thalamus, hypothalamus), brainstem, or spinal cord (Fremeau et al., 2001, 2004a; Herzog et al., 2001; Kaneko and Fujiyama, 2002; Varoqui et al., 2002). Footpad inflammation was previously seen to increase glutamate release in the LC (Nonaka et al., 2017). Furthermore, glutamate stimulates noradrenaline release in the SC by activating LC AMPA (a-amino-3-hydroxy-5-methylisoxazole-4-propionic acid) receptors (Singewald and Philippu, 1998), suggesting that LC glutamatergic activation is caused by acute nociceptive input. Accordingly, increased NMDAR1 activity and pCAMKII are observed in the LC of CCI7d rats following a noxious stimulus (Alba-Delgado et al., 2012b). We previously demonstrated that 7 days of neuropathy increases PGi activation (Alba-Delgado et al., 2012a), the source of the main glutamatergic afferents to the LC that expresses VGLUT2. This suggests that the PGi could be responsible for the increase in the excitatory marker observed in CCI7d rats, which would be consistent with the increase in excitatory synaptic transmission in LC neurons seen previously, 7 days after CCI (Rohampour et al., 2017). On the other hand, an increase in noradrenergic immunoreactivity in the spinal dorsal horn was reported 2 days and 2 weeks after CCI (Ma and Eisenach, 2003). Together, these data might suggest that short-term nerve injury affects excitatory synaptic markers significantly in LC neurons, which could imply a gradual enhancement in the noradrenergic system as an adaptive mechanism to counteract chronic pain transmission.

It was also reported previously that SNL rats increase basal GABA release and the immunoreactivity for the GABA-synthesizing enzyme GAD in the LC 5 days after SNL (Yoshizumi et al., 2012), suggesting increased GABA tone after early nerve injury. Similarly, we did not find differences in the density of VGAT^+^ puncta 7 or 28 days after neuropathy. Our previous studies failed to detect differences in the expression of gephyrin 14 days after neuropathy, a protein selectively located at inhibitory synapses in the LC (Bravo et al., 2013). This discrepancy could be due to the different animal models employed, as different approaches to induce neuropathy may promote similar changes in the LC associated with the instauration of neuropathy but these events could occur over different timescales.

When exploring long-term CCI (CCI28d), we did not find any significant effect on the density of VGLUT1^+^ puncta. However, evaluating the complete LC region, revealed a decrease in VGLUT2 density without producing a net difference in the VGLUT2/VGAT ratio. Regarding noradrenergic perisomatic study, CCI28d showed a decrease in VGLUT2 density accompanied by a decrease in the ratio VGLUT2/VGAT when compared to CCI7d. That is, short-term neuropathic pain causes an increase in excitatory synaptic markers that falls to normal levels (Sham) in association with long-term pain. In mice, an increase in the VGLUT2 density during early spared nerve injury was also followed by a return to control levels in pain-related brain areas (Wang et al., 2015). Moreover, long-term peripheral nerve injury augments extracellular glutamate by down-regulating GLT-1, thereby inhibiting evoked glutamate release in the LC *via* the activation of mGluRs. This response would dampen the neuronal activity evoked in the LC in response to a noxious input, which is important when contemplating pain-induced endogenous analgesia (Kimura et al., 2015). These data suggest that the endogenous protective analgesia mediated by the LC may become exhausted. We did not find differences in the density of VGAT at this time point after nerve injury, consistent with previous studies that failed to find any change in GAD immunoreactivity in SNL-6W rats (Yoshizumi et al., 2012). Importantly, the differences in the VGLUT2/VGAT ratio at both CCI7d and CCI28d are mainly due to changes in the dorsal LC as no significant differences were evident in the ventral domain. These findings suggest a topographic distribution in the LC. We recently reported that LC neurons located specifically in the dorsal domain are activated (expressing c-Fos) at early times post-injury but not after long-term injury (Llorca-Torralba et al., 2022). Furthermore, chemogenetic inactivation of LC-SC neurons clearly increases pain sensitivity 2 days after nerve injury. This effect is reduced at 7 days and it is lost at 30 days after neuropathic pain induction (Llorca-Torralba et al., 2022). Although discrepancies might occur between studies regarding the anatomical denomination of the dorsal and ventral domains, and different rat strains were used (Sprague-Dawley vs. Long-Evans rats), these findings suggest that specific subpopulations of noradrenergic neurons mediate pain transmission.

Considering our previous findings following long-term neuropathy, we also evaluated the size of TH^+^ cell somas. Although we did not find any significant differences in soma size among nerve-injured animals, there were differences in the frequency distribution of soma sizes, with a higher percentage of neurons with a soma of 150 μm^2^ after long-term pain. Chronic stress also produces alterations in soma size, but in the opposite direction. Hence, TH^+^ cells had a smaller soma in chronic mild stress (CMS) animals, an effect that was further exaggerated in CCI-CMS rats. Additional ultrastructural studies in long-term pain animals showed the presence of dilated ER and mitochondria, as well as a decrease in the density of mitochondria and lysosomes. These data are consistent with the idea that a loss of mitochondria impairs lysosomal activity (Demers-Lamarche et al., 2016). Mitochondria play a central role in neuron survival and death, and a loss of these structures might mean a reduction in the available energy substrates, which could hinder a cell's ability to withstand insults. Although there is no data about the ultrastructural changes in the LC of neuropathic rats, selective destruction of noradrenergic neurons using the neurotoxins 6-OHDA or DSP-4 induces depressive-like behavior (Szot et al., 2016) and impairs the emotional component of pain (Bravo et al., 2016), classic behaviors related to long-term but not short-term pain.

Interestingly, in animal models of neurodegenerative disorders, the shrinkage of TH^+^ cells in the LC has often been described (German et al., 2005; Polak et al., 2011). Also, alterations to mitochondrial function and other microstructural changes in the LC, as well as degeneration of noradrenergic axons, have been demonstrated in the frontal cortex of rats subjected to long-term stress (Kitayama et al., 1997, 2008). An increasing number of studies have reported that mitochondrial activities (apoptosis, oxidative stress, etc.) are altered in anxiety and mood disorders, and it has been suggested that individuals with mitochondrial dysfunction would be vulnerable to stress and psychiatric disorders (Hollis et al., 2015; Filiou and Sandi, 2019). Thus, our findings invite an exploration of mitochondrial function in chronic neuropathic pain models.

We also explored the effect of nerve injury on the number of CC3^+^ cells in the LC. Increased activation of CC3 is considered a marker of impending neuronal death (Thornberry and Lazebnik, 1998) but also plays non-apoptotic roles related to neuroadaptation, synapse refinement (D'Amelio et al., 2010; Fan et al., 2014), and astrogliosis (Aras et al., 2012). Regarding CC3^+^ cells, there was a significant increase in the CCI28d LC but such increase was found in DBH^−^ cells, indicating that noradrenergic neurons were not involved. Indeed, most CC3 expression corresponds to glial GFAP^+^ cells in spite of GFAP staining did not change among groups. Interestingly, the pattern of CC3 has been detected largely in non-neuronal cell populations without matching with TUNEL death marker (Stevenson et al., 2018). Also, rats submitted to a protocol of brain excitotoxic damage showed astroglial cells positive for CC3 without associated apoptotic death (Acarin et al., 2007). Moreover, CC3 upregulation occurs in absence of cell death in both *in vitro* and *ex vivo* models of astrogliosis (Aras et al., 2012). In this study, the inhibition of CC3 reduced the expression of proteins associated with astrogliosis (glutamine synthetase and fibroblast growth factor-2) without altering astrocytes reactive morphology. Thus, one possibility is that the impact of suffering by LC in response to neuropathic pain activates the CC3 expression in astrocytes as a remodeling process but not of cell death.

Other studies demonstrated that CC3 can be activated *via* the mitochondrial pathway by stimulating NMDA receptors, without causing cell death (Li et al., 2010). Indeed, local activation of CC3 by photostimulation of mitochondria-targeted KillerRed, which triggers mitochondrial damage induces local spine elimination and dendrite retraction in cultured hippocampal neurons, without inducing full apoptosis (Erturk et al., 2014). Interestingly, we have found an important affectation of the excitatory/inhibitory ratio in the LC of CCI7d that is lost at CCI28d when CC3 is increased in glial cells. These results suggest once again that CC3 should be not considered a marker of death cell but rather a remodeling process in response to early glutamate overstimulation or *via* mitochondria damage.

In summary, this study highlights the importance of considering remodeling process occurring in the LC cells of neuropathic pain animals in a time-dependent manner. First, short-term pain affects excitatory neurotransmission in the LC, probably as an adaptive mechanism against the instauration of chronic pain. Second, long-term pain might induce ultrastructural changes in LC, which activates astroglial CC3 expression as a possible adaptive mechanism that might contribute to LC plasticity in long-term pain. Consequently, these phenomena might drive the negative emotional impact underlying chronic neuropathic pain.

## Data availability statement

The raw data supporting the conclusions of this article will be made available by the authors, without undue reservation.

## Ethics statement

The animal study was reviewed and approved by Committee for Animal Experimentation at the University of Cadiz.

## Author contributions

EB, JN, and LB designed the study and wrote the manuscript. ML-T performed the behavioral experiments. PM and LB performed the tissue extraction and processing, they designed the protocols for immunohistochemistry, and as well as performed the acquisition and analysis of the data. JL-C contributed to the processing of tissue and the image analysis. EB and JN supervised the study. All authors revised and approved the submitted version of the manuscript.

## Funding

This study was supported by grants co-financed by the Fondo Europeo de Desarrollo Regional (FEDER)-UE (A way to build Europe) from the Ministerio de Economía y Competitividad (MINECO: RTI2018-099778-B-I00) and the Ministerio de Salud-Instituto de Salud Carlos III (PI18/01691), by the Consejería de Salud de la Junta de Andalucía (PI-0134-2018; Grant No. P20-00958), by the Programa Operativo de Andalucía FEDER, Iniciativa Territorial Integrada ITI 2014-2020 Consejería Salud, Junta de Andalucía (PI-0080-2017), by the Instituto de Investigación e Innovación en Ciencias Biomédicas de Cádiz (INiBICA LI19/06IN-CO22; IN-C09), by the Consejería de Economía, Innovación, Ciencia y Empleo de la Junta de Andalucía (CTS-510), and by the “CIBERSAM”: CIBER-Consorcio Centro de Investigación Biomédica en Red- (CB07/09/0033), Instituto de Salud Carlos III, Ministerio de Ciencia e Innovación and the European Union's Horizon 2020 Research and Innovation Programme under the Marie Sklodowska-Curie grant agreement (Grant No. 955684). Figures were created with BioRender.com.

## Conflict of interest

The authors declare that the research was conducted in the absence of any commercial or financial relationships that could be construed as a potential conflict of interest.

## Publisher's note

All claims expressed in this article are solely those of the authors and do not necessarily represent those of their affiliated organizations, or those of the publisher, the editors and the reviewers. Any product that may be evaluated in this article, or claim that may be made by its manufacturer, is not guaranteed or endorsed by the publisher.
